# A Labor Saver

**Published:** 1900-11

**Authors:** 


A LABOR SAVER.—Several of our exchanges constantly do us the honor—but without giving the "Red-Back" credit—of clipping our news items and editorial notes. That's all right. It is much easier to clip than to think and write. But this way of earning their bread by the sweat of *our brow,* you know. Well, it saves our esteemed contemporaries' brows, and as we are something of a humanitarian I reckon we ought n't to kick. Let it go, if they think we can do these things better than they can. The "Red-Back" is a labor saver—for the other fellow.

---

THE BUILDING of M. J. Breitenbach Co., 100 Warren Street, New York, was totally destroyed by fire on Monday, October 28th (ult.). With true American spirit they didn't allow "a little thing like that" to set them back. In less than ten days they were on their feet again. The market being well supplied with their excellent product *Pepto-Mangan—Gude* there was and will be no interruption of their business. Fire may come and explosions may blow; but like Tennyson's "Brook," *Pepto-Mangan—Gude* goes on forever. We learn that Mr. Breitenbach and Mr. Wells, of the firm, and also Mr. Main, of Tarrant & Co., had a pretty close call. Just as these gentlemen left the building it was blown to kingdom come. The JOURNAL offers its sincerest congratulations on their escape.

